# Pre-hospital symptoms associated with acute bacterial meningitis differs between children and adults

**DOI:** 10.1038/s41598-023-48161-x

**Published:** 2023-12-06

**Authors:** Nichlas Hovmand, Helle Collatz Christensen, Lene Fogt Lundbo, Gitte Kronborg, Perle Darsø, Stig Nikolaj Fasmer Blomberg, Thomas Benfield

**Affiliations:** 1 Center for Research & Disruption of Infectious Diseases (CREDID), Department of Infectious Diseases, Copenhagen University Hospital–Amager and Hvidovre, Kettegaard Alle 30, 2650 Hvidovre, Denmark; 2 Department of Clinical Medicine, Faculty of Health and Medical Sciences, University of Copenhagen, Blegdamsvej 3B, 2200 Copenhagen N, Denmark; 3 Emergency Medical Services, Capital Region of Denmark, Telegrafvej 5, 2750 Ballerup, Denmark; 4 Department of Infectious Diseases, Copenhagen University Hospital–Amager and Hvidovre, Kettegaard Alle 30, 2650 Hvidovre, Denmark; 5 Center for Health, Capital Region of Denmark, Kongens Vænge 2, 3400 Hillerød, Denmark

**Keywords:** Fatigue, Fever, Neurological manifestations, Meningitis

## Abstract

Community acquired bacterial meningitis (CABM) is a medical emergency requiring timely appropriate action. More knowledge about pre-hospital symptoms is needed. Retrospective observational study of pre-hospital management in patients with CABM between 2016 and 2021 admitted to a hospital in the Capital Region of Denmark. Reported symptoms were extracted from archived audio files of the initial phone call to emergency medical service. The majority of the 209 patients (82%) were adults. The most common symptoms were altered mental state (58%) and fever (57%), while neck stiffness was less common (9%). Children more often presented with fever, fatigue, rashes, and neck stiffness, while adults more often presented with altered mental state, and leg pain. Most patients (85%) reported at least 1 of the 3 symptoms in the classical triad of meningitis, while 3% reported all 3. Children more often presented at least 2 of 3 symptoms in the triad. One child (3%) and 7 adults (4%) received antibiotics pre-admission. Patients with CABM reported a variety of symptoms that differed significantly in children and adults. The classic triad was rare. Very few patients received antibiotics pre-admission. We suggest that questioning relevant symptoms should be done in febrile or mentally altered patients.

## Introduction

Meningitis can be difficult to diagnose as patient history and clinical examination alone can neither confirm nor exclude the diagnose with certainty^1^. Community acquired bacterial meningitis (CABM) remains a medical emergency of global significance that often leads to an unfavorable outcome, even though appropriate treatment is widely available^2,3^. CABM is classically characterized by a triad of symptoms: fever, neck stiffness, and altered mental status. However, the triad is present in less than 50% of patients^4^. Specific symptoms of meningitis, e.g. headache and neck stiffness, are less prominent in CABM compared to viral meningitis, and symptoms in the initial phases of CABM are unspecific^4,5^. Patients who are not diagnosed early can present with symptoms mimicking a wide variety of both infectious and non-infectious medical conditions^6^. High age, comorbidities, male sex and delayed treatment are well known risk factors for poor outcome from CABM and it is important to raise suspicion and initiate appropriate antibiotic treatment quickly, as the infection can progress from mild symptoms to a life-threatening condition in a matter of hours^7–11^.

The residents in the Capital Region of Denmark have 2 available phone numbers to emergency medical services (EMS). One number is for absolute medical emergencies, and it receives 130,000 health related calls annually. The second number is a medical helpline staffed by registered specialist nurses that receives 950,000 calls annually. The regional guideline for pre-hospital handling of patients suspected of CABM is to immediately send an ambulance and a medical doctor to the patient, and if CABM is suspected on-site, antibiotics should be administered immediately.

Previous studies of early symptoms in patients with CABM mainly reported symptoms in infants and/or in hospital settings after admission^11–15^. More knowledge about the earliest presentations of CABM in all age groups may help to improve future care. We recently published a study showing that nonspecific symptoms dominate at first contact to emergency healthcare services among patients with invasive meningococcal disease^16^.

Here we present symptoms reported at the very first contact to EMS for patients with CABM, as we aim to explore how these patients can be diagnosed in the earliest stages of the disease.

## Methods

### Study design

In the Capital Region of Denmark, with a population of 1,800,000, a retrospective observational study of patients with CABM between January 1st of 2016 and December 31st of 2020 was conducted.

As this was a quality assessment project, approval by the Committee on Health Research Ethics was not required. The need for informed consent for this study was waived by Center for Health and the Emergency Medical Services in the Capital Region of Denmark who gave permissions to collect, analyze, and publish data as required by Danish legislation^17^. All methods were performed in accordance with the relevant guidelines and regulations.

### Patient identification and data collection

Potential patients were identified by diagnosis codes for CABM during hospital admissions (International Classification of Disease, 10th edition, (ICD-10) codes: DG00*) or IMD (ICD-10: DA39*). To include potential patients who were not given a diagnosis code, patients with either molecular detection, visualization, or culture of bacterial pathogens from cerebrospinal fluid or identification of *N. meningitidis* in blood were identified in the region’s databases of microbiology. A physician (NH) reviewed the electronic health records (EHR) of all patients to determine which patients had CABM. Patients with CABM who had contact to EMS prior to hospitalization were included.

Each patient’s initial phone call to EMS was recorded and listened to by a physician (NH) who evaluated which symptoms were asked about or mentioned, and which symptoms were confirmed to be present. Some symptoms were grouped, e.g., altered mental state covers a spectrum from mild confusion to unconsciousness. Data on pre-hospital initiation of antibiotics were collected from ambulance personnel registrations. Data on further patient characteristics, hospital handling, and 30-day vital status was collected from EHR. Appropriate treatment was defined as treatment against a known etiology or empirical treatment for suspected bacterial meningitis as per local guideline.

### Statistical analysis

Two-sided *P*-values of less than 0.05 were considered statistically significant and were not adjusted for multiple testing due to the explorative nature of this study. Fisher’s Exact Test was used to calculate *P*-values between groups. Patients were grouped as either children under 18 years of age or adults. Because patients were not asked about all symptoms, analyses were performed for both patients reporting a symptom of patients asked about the symptom and of patients reporting a symptom of all patients. Spearman's rank correlation coefficient was used to calculate paired correlations. Median and interquartile range or proportion and percentage was used as appropriate. R version 3.6.0 was used for statistical analyses.

### Ethics approval and consent to participate

As this was a quality assessment project, approval by the Committee on Health Research Ethics was not required. The need for informed consent for this study was waived by Center for Health and the Emergency Medical Services in the Capital Region of Denmark who gave permissions to collect, analyze, and publish data as required by Danish legislation^17^. All methods were performed in accordance with the relevant guidelines and regulations.

## Results

### Patient characteristics

**Figure 1:**
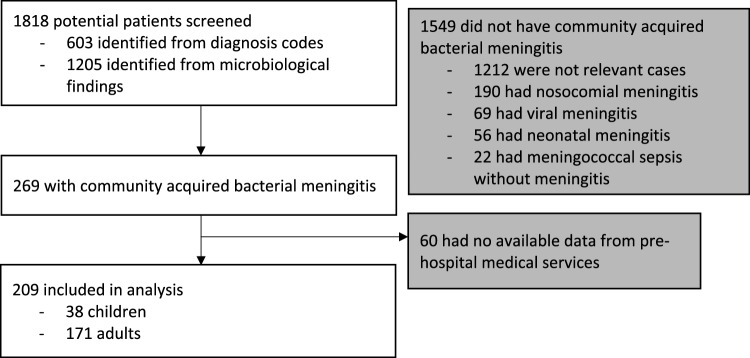
Flow chart of patient identification, inclusion, and exclusion. This flow chart illustrates the sources that patients were identified from and reasons for exclusion. Following this process, 209 patients with community acquired bacterial meningitis who had contact to emergency medical services prior to hospitalization between 2016 and 2021 in the Capital Region of Denmark were included in analysis.

We included 209 patients for analysis from 1818 patients who were individually screened based on either ICD-10 codes or the clinical microbiological databases (Fig. 1). We identified 269 patients with CABM, of whom 60 did not have a pre-hospital contact.

The majority were adults ≥ 18 years of age (171 (82%)). The median age was 61 years, and 47% were female (Table 1). The patients were evenly distributed over the 5-year period. The overall most common single bacterial etiology was *Streptococcus pneumoniae* (38%), however, children were more likely to have a meningococcal etiology compared to adults (32% vs 5%, *P* < 0.01) (Supplementary Fig. 1). The medical helpline accounted for 58% of calls, while the emergency call center accounted for 42%. Contact to the medical helpline was more frequent for children than for adults (89% vs 51%, *P* < 0.01). Of the 209 patients, 8 (4%) received pre-hospital antibiotics. Thirty-day mortality was 11% (children 5% vs. adults 12%, *P* = 0.26). There were no significant differences between children and adults by sex, year of disease, or use of pre-hospital antibiotics.

**Table 1:** Patient characteristics.

	All N = 209	Age < 18 years N = 38	Age ≥ 18 years N = 171	*P*-value
Age (years)				–
Median (IQR)	61 (38;73)	0 (1;6)	66 (53;76)	
Gender				0.85
Female	99 (47%)	17 (45%)	82 (48%)	
Male	110 (53%)	21 (55%)	89 (52%)	
Bacterial etiology				
Pneumococcal	80 (38%)	12 (32%)	68 (40%)	0.46
Meningococcal	21 (10%)	12 (32%)	9 (5%)	< 0.01
Other	93 (44%)	13 (34%)	80 (47%)	0.21
Unknown	15 (7%)	1 (3%)	14 (8%)	0.32
Year of disease				
2016	38 (18%)	5 (13%)	33 (19%)	0.49
2017	33 (16%)	6 (16%)	27 (16%)	1.00
2018	37 (18%)	6 (16%)	31 (18%)	0.82
2019	31 (15%)	8 (21%)	23 (13%)	0.31
2020	39 (19%)	8 (21%)	31 (18%)	0.65
2021	31 (15%)	5 (13%)	26 (15%)	1.00
Emergency service used				< 0.01
Emergency call center	88 (42%)	4 (11%)	84 (49%)	
Medical helpline	121 (58%)	34 (89%)	87 (51%)	
30-day mortality				0.26
Survivor	186 (89%)	36 (95%)	150 (88%)	
Non-survivor	23 (11%)	2 (5%)	21 (12%)	
Pre-hospital antibiotic				1.00
Yes	8 (4%)	1 (3%)	7 (4%)	
No	201 (96%)	37 (97%)	164 (96%)	

List of patient characteristics of the 209 patients with community acquired bacterial meningitis who had available data from emergency medical services prior to hospitalization between 2016 and 2021 in the Capital Region of Denmark. Patients were grouped as either children under 18 years of age or adults.

### Symptoms at initial phone call to EMS

A total of 21 different symptoms were mentioned in the 209 initial phone calls to EMS. On average, 3.6 symptoms were reported to be present by each caller, and 4.8 symptoms were asked about or mentioned in each phone call (Table 2). The most common symptom was altered mental state (present in 58% (121 of 209) of patients). The other most frequently present symptoms were fever (57%), fatigue (53%), headache (33%), and vomiting (25%). Children were more likely to present with fever (92% (35 of 38) of children vs. 61% (105 of 171) of adults, *P* < 0.01), fatigue (71% vs. 49%, *P* = 0.01), rash (16% vs. 1%, *P* < 0.01), or neck stiffness (18% vs. 6%, *P* = *0.02*), while adult patients were more likely to present with altered mental state (42% vs. 61%, *P* = 0.04) or leg pain (3% vs. 15%, *P* = 0.05).

**Table 2:** Symptoms at initial call to emergency medical services.

	Symptom present/symptom asked about			*P*-value	
	All patients N = 209	Age < 18 years N = 38	Age ≥ 18 years N = 171	Present of asked about^A^	Present of all patients^B^
Altered mental state	121/142	16/30	105/112	< 0.01	0.04
Fever	120/133	35/37	85/96	0.51	< 0.01
Fatigue	110/115	27/30	83/85	0.11	0.01
Headache	70/74	8/11	62/63	0.01	0.09
Vomiting	53/64	13/18	40/46	0.27	0.21
Ear pain	40/40	5/5	35/35	1.00	0.37
Dyspnea	34/66	5/12	29/54	0.53	0.81
Common cold	27/35	8/12	19/23	0.40	0.11
Leg pain	26/31	1/2	25/29	0.30	0.05
Tremors and/or seizures	24/25	4/4	20/21	1.00	1.00
Back pain	21/21	1/1	20/20	1.00	0.13
Diarrhea	18/24	5/9	13/15	0.15	0.33
Stiffness of the neck	18/36	7/12	11/22	0.73	0.02
Coughing	13/26	1/4	12/22	0.59	0.47
Palsy	12/37	0/0	12/37	1.00	0.13
Abdominal pain	8/12	3/5	5/7	0.07	0.16
Sore throat	8/18	2/5	6/13	1.00	0.64
Sparse urination	8/23	3/15	5/8	0.07	0.16
Rash and/or petechiae	7/45	6/2	1/23	0.05	< 0.01
Photophobia	4/4	2/2	2/2	1.00	0.15
Chest pain	4/11	0/0	4/11	1.00	1.00

List of symptoms mentioned in 209 initial phone calls to emergency medical services. Eighty-eight calls were to the emergency call center, while 121 calls were to the medical helpline. For any symptom, it was registered in how many calls the symptom was present and in how many calls the symptom was asked about and/or mentioned. Because all symptoms were not always asked about, *P-*values were calculated for both patients with symptoms present of patients asked about symptoms and of all patients.

^A^P values for comparisons between children and adults for the ratio of patients reporting a symptom of the patients asked about the symptom.

^B^P values for comparisons between children and adults for the ratio of patients reporting a symptom of all patients whether they were asked about the symptom or not.

**Figure 2:**
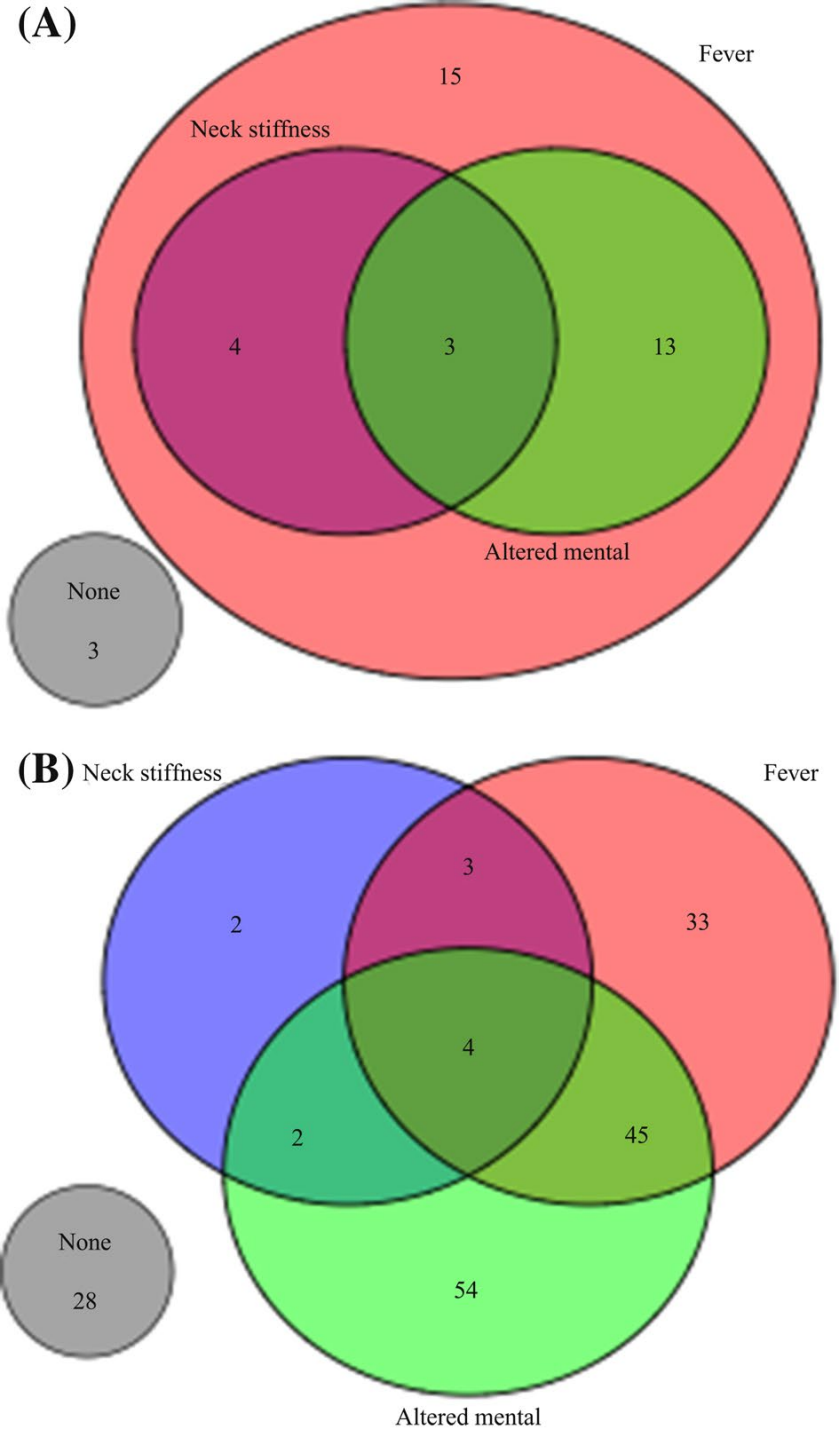
Venn diagram of triad of symptoms for the 38 children (**A**) and 171 adults (**B**). Venn diagram showing relation between the 3 symptoms of the classical triad of meningitis for the 38 children (**A**) and 171 adults (**B**) with community acquired bacterial meningitis who had contact to emergency medical services prior to hospitalization between 2016 and 2021 in the Capital Region of Denmark.

**Figure 3:**
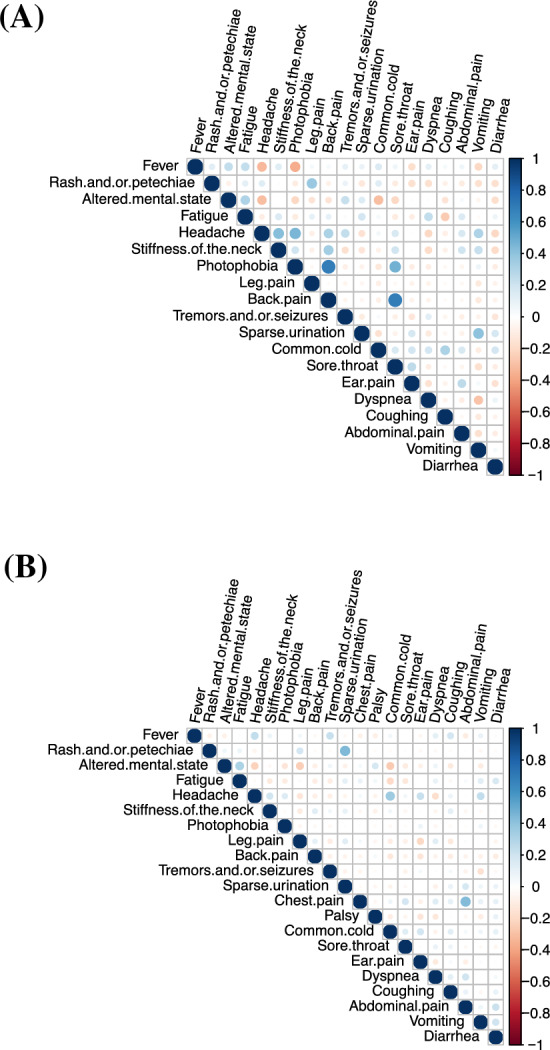
Paired correlations between symptoms for the 38 children (**A**) and 171 adults (**B**). Plot showing paired correlations between symptoms at first contact to emergency medical services for the 38 children (**A**) and 171 adults (**B**) with community acquired bacterial meningitis who had contact to emergency medical services prior to hospitalization between 2016 and 2021 in the Capital Region of Denmark. The highest correlations for children (**A**) were seen between photophobia and back pain (r = 0.70, CI95%: [0.49;0.83], *P* < 0.01), back pain and sore throat (r = 0.70, CI95%: [0.49;0.83], *P* < 0.01), and photophobia and sore throat (r = 0.47, CI95%: [0.18;0.69], *P* < 0.01). The highest correlations for adults (**B**) were seen between rashes and sparse urination (r = 0.44, CI95%: [0.31;0.56], *P* < 0.01), chest pain and abdominal pain (r = 0.43, CI95%: [0.30;0.55], *P* < 0.01), headache and common cold (r = 0.35, CI95%: [0.21;0.48], *P* < 0.01), and altered mental state and fatigue (r = 0.31, CI95%: [0.17;0.44], *P* < 0.01).

Only 7 (3%) of the 209 patients reported all three symptoms of the classical meningitis triad (Fever, neck stiffness, and altered mental status) to be present (3/38 (8%) children vs. 3/171 (2%) adults, *P* = 0.08) (Fig. 2, Supplementary Fig. 2). Seventy-four (35%) presented with at least 2 of the 3 symptoms (20/38 (53%) children vs. 54/171 (32%) adults, *P* = 0.02), while 178 (85%) reported at least 1 of the 3 symptoms (35/38 (92%) children vs. 143/171 (84%) adults, *P* = 0.22). The highest paired correlations for children were seen between photophobia and back pain (r = 0.70, CI95%: [0.49;0.83], *P* < 0.01), back pain and sore throat (r = 0.70, CI95%: [0.49;0.83], *P* < 0.01), and photophobia and sore throat (r = 0.47, CI95%: [0.18;0.69], *P* < 0.01). The highest correlations for adults were seen between rashes and sparse urination (r = 0.44, CI95%: [0.31;0.56], *P* < 0.01), chest pain and abdominal pain (r = 0.43, CI95%: [0.30;0.55], *P* < 0.01), headache and common cold (r = 0.35, CI95%: [0.21;0.48], *P* < 0.01), and altered mental state and fatigue (r = 0.31, CI95%: [0.17;0.44], *P* < 0.01) (Fig. 3, Supplementary Fig. 3).

### Pre-hospital management

**Figure 4:**
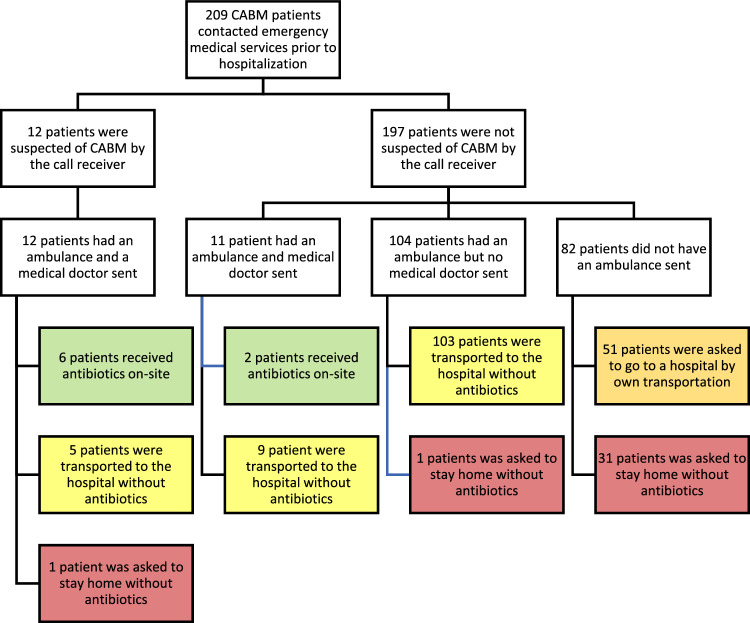
Flow chart of pre-hospital management of patients with community acquired bacterial meningitis. Two hundred and nine patients who had community acquired bacterial meningitis (CABM) called emergency medical services prior to hospitalization. This flow chart illustrates how patients were handled prehospitally. Eight of the 209 patients received antibiotics on-site after evaluation from a medical doctor as per the region’s guideline (green). One hundred and seventeen patients were transported to a hospital by an ambulance but did not receive treatment (yellow). Fifty-one patients were asked to go to the hospital by own transportation (orange). Thirty-three patients were asked to stay home (red). All 209 patients were hospitalized by one way or another within 71 h. Median time from initial contact to hospitalization was 1.2 h (IQR 0.9 to 2.4 h).

Patients were suspected of CABM in 12 (3 children and 9 adults) of the 209 phone calls (Fig. 4). All 12 had an ambulance and a medical doctor team sent immediately; 6 received antibiotic treatment on-site. Five were taken to the hospital without antibiotic treatment, while 1 was asked to remain at home.

Of the 197 patients who were not suspected of CABM during the EMS call, 2 received antibiotic treatment on-site, while 112 patients were taken to the hospital by an ambulance without receiving antibiotic treatment on-site, 51 were advised to go to the hospital by themselves, and 32 patients were advised to remain at home.

Regardless, all 209 patients were hospitalized at a maximum of 71 h from the initial EMS call: 189 within 24 h and 20 later than 24 h after the initial EMS call. Median time from initial contact to hospitalization was 1.2 h (children 1.8 h and adults 1.2 h). Children were more likely to be asked to stay home (13/38 (34%) children vs. 18/171 (11%) adults, *P* < 0.01) and less likely to have an ambulance sent to them compared to adults (8/38 (21%) children vs. 119/171 (69%) adults, *P* < 0.01) (Supplementary Fig. 4).

### Hospital management and outcome

**Figure 5:**
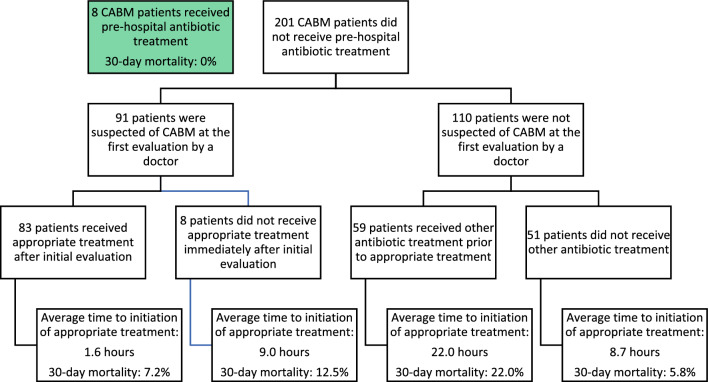
Flow chart of hospital management of patients with community acquired bacterial meningitis. Two hundred and one patients who had community acquired bacterial meningitis (CABM) arrived at a hospital without being treated prehospitally. Ninety-one were suspected of CABM at first evaluation of which 83 were treated as such. One hundred and ten patients were not suspected of CABM at first evaluation. Fifty-nine of these initially received other antibiotic treatment while 51 did not receive other antibiotic treatment before initiation of appropriate treatment. Appropriate treatment was defined as treatment against a known etiology or empirical treatment as per local guideline for suspected bacterial meningitis.

Eight patients received pre-hospital antibiotic treatment and none (0%) of them died within 30 days (Fig. 5). Of the 201 patients who did not receive antibiotic treatment prehospitally, 91 were suspected of CABM at the initial assessment by a doctor upon admission to hospital, of which 83 were treated as such, whereof 6 (7.2%) died within 30 days. One (12.5%) of the 8 patients who were suspected of CABM but not treated immediately died within 30 days. One-hundred-and-ten patients were not suspected of CABM at the first doctor’s evaluation. Fifty-nine of those patients received other antibiotic treatment prior to appropriate treatment, and 13 (22.0%) of them died within 30 days. Fifty-one did not receive other antibiotic treatment, and 3 (5.5%) of them died within 30 days.

There was no difference between children and adults regarding how often they were suspected of CABM prehospitally or at initial evaluation [20/38 (53%) children vs. 79/171 (46%) adults, *P* = 0.48] (Supplementary Fig. 5).

## Discussion

Very few patients with CABM were identified and treated as such in the pre-hospital setting. The most common symptoms were symptoms known to be associated with CABM, e.g., altered mental state, fever, fatigue, headache, and vomiting. Children were more likely to present with fever, fatigue, rashes, and neck stiffness, while adult patients were more likely to present with altered mental state, headache, and leg pain. Most patients presented with at least 1 of the 3 symptoms in the classical triad of meningitis, but very few presented with all 3. Children more often reported at least 2 of 3 symptoms in the triad. Compared to children, adults were more likely to call the emergency call center, to have an ambulance sent to them and less likely to be asked to stay at home, suggesting that adults were more frequently severely ill at initial contact to EMS. This could be due to children being watched by parents who would react to a progression of the childrens symptoms.

The current literature about pre-hospital symptoms in patients with meningitis is sparse and based on either hospital records or parental recall^18,19^. To our knowledge this is the first study to examine pre-hospital presentations of patients with CABM from recorded pre-hospital phone calls.

To optimize early management of patients with CABM it is vital to raise the suspicion of CABM even when the classic triad of symptoms is absent. Overlapping symptoms between several acute medical conditions is a likely contributor of failure to identify individuals with CABM early. Additionally, many of the medical conditions are more common than CABM, and thus, likely to receive higher priority than less common conditions. An attempt to create a system or line of questioning with the sensitivity to identify all CABM immediately would be highly unspecific and would delay relevant care for patients with other time sensitive medical emergencies, e.g., stroke. However, evidence guided focused questioning could make it easier to distinguish the different diagnoses and thus identify patients with CABM earlier even when meningeal symptoms are absent.

As most patients reported at least 1 symptom from the triad, we suggest that all patients with either fever, altered mental state, or neck stiffness should be asked about or examined for all 3 symptoms. We suggest that CABM is considered in all febrile or mentally altered patients until another cause of the symptoms have been established. Special care to not miss diagnosing CABM should be shown in children with fatigue, rash, and/or neck stiffness and in adults with altered mental state, headache, and/or leg pain. In any patient suspected of CABM, immediate action must be taken to ensure immediate appropriate care including lumbar puncture and initiation of antibiotic treatment.

## Limitations

A limitation of this study is that patients were not asked about all symptoms and that some symptoms were grouped, representing a spectrum of severity of the symptom. This limits the study’s ability to examine the complete spectrum of symptoms. It is also a limitation that the diagnosis cannot be ruled in nor out at the time of the initial contact. However, we believe that the knowledge about the patients' earliest presentations are relevant because future patients with meningitis will have the same presentations in similar settings.

The study is further limited by its retrospective nature and the fact that pre-hospital data were missing for 60 of 269 patients with CABM which were likely missing because their family practitioner send them to the hospital without involving the EMS or they went to the hospital by themselves without contacting EMS.

## Conclusions

Patients with CABM are rarely diagnosed in the pre-hospital setting. We were able to examine data from pre-hospital phone calls to EMS for 209 patients with confirmed CABM, allowing us to report unique knowledge on early symptoms in CABM for patients of all ages. We found that only 4% of the patients were treated for CABM in the pre-hospital setting. Most patients were febrile or mentally altered and that symptoms varied between children and adults. Thus, we suggest that all febrile or mentally altered patients should be suspected for CABM and that age specific questioning of relevant symptoms should be used to either raise or lower the suspicion.

More research is needed to gain further knowledge about which patients with non-specific symptoms should be suspected of CABM to optimize early management of the disease.

### Supplementary Information

Supplementary Figures.

## Data Availability

All data generated or analyzed during this study are included in this published article.

## Abbreviations

CABM: Community acquired bacterial meningitis
EHR: Electronic health record
EMS: Emergency medical services
