# Supplementary material for: Pre-hospital symptoms associated with acute bacterial meningitis differs between children and adults
Source: Sci Rep. 2023 Dec 6;13:21479. doi: 10.1038/s41598-023-48161-x (PMC10697940; doi:10.1038/s41598-023-48161-x)
Supplement: Supplementary file 1 — Supplementary Figures. [file 41598_2023_48161_MOESM1_ESM.docx]

**Supplementary Figures**

**Supplementary Figure 1:** Pathogens causing community acquired bacterial meningitis.

| Pathogen | All  N = 209 | Children <18  N = 38 | Adults >18  N = 171 |
| --- | --- | --- | --- |
| \| Streptococcus Pneumoniae \| \| --- \| \| Haemophilus influenzae \| \| Neisseria Meningitidis \| \| Staphylococcus aureus \| \| Unknown \| \| Listeria \| \| Streptococcus grp. A \| \| E. coli \| \| Streptococcus dysgalactiae \| \| Streptococcus anginosus \| \| Streptococcus bovis \| \| Streptococcus mitis \| \| Streptococcus grp. B \| \| Acinetobacter lwoffii \| \| Aggregatibacter aphrophilus \| \| Fusobacterium necrophorum \| \| Klebsiella pneumoniae \| \| Lactobaccilus salivarius \| \| Pseudomonas aeruginosa \| \| Staphylococcus warneri \| \| Streptococcus oralis \| \| Streptococcus salivarius \| \| Streptococcus species \| | \| 80 \| \| --- \| \| 22 \| \| 21 \| \| 15 \| \| 15 \| \| 11 \| \| 8 \| \| 7 \| \| 6 \| \| 4 \| \| 4 \| \| 3 \| \| 3 \| \| 1 \| \| 1 \| \| 1 \| \| 1 \| \| 1 \| \| 1 \| \| 1 \| \| 1 \| \| 1 \| \| 1 \| | \| 12 \| \| --- \| \| 6 \| \| 12 \| \| 0 \| \| 1 \| \| 1 \| \| 4 \| \| 0 \| \| 0 \| \| 0 \| \| 0 \| \| 0 \| \| 1 \| \| 0 \| \| 0 \| \| 1 \| \| 0 \| \| 0 \| \| 0 \| \| 0 \| \| 0 \| \| 0 \| \| 0 \| | \| 68 \| \| --- \| \| 16 \| \| 9 \| \| 15 \| \| 14 \| \| 10 \| \| 4 \| \| 7 \| \| 6 \| \| 4 \| \| 4 \| \| 3 \| \| 2 \| \| 1 \| \| 1 \| \| 0 \| \| 1 \| \| 1 \| \| 1 \| \| 1 \| \| 1 \| \| 1 \| \| 1 \| |

**Supplementary** **Figure 2:** Venn diagram of triad of symptoms for all 209 patients.

Venn diagram showing relation between the 3 symptoms of the classical triad of meningitis for the 209 patients with community acquired bacterial meningitis who had contact to emergency medical services prior to hospitalization between 2016 and 2021 in the Capital Region of Denmark.

**Supplementary** **Figure 3:** Paired correlations between symptoms for all 209 patients.


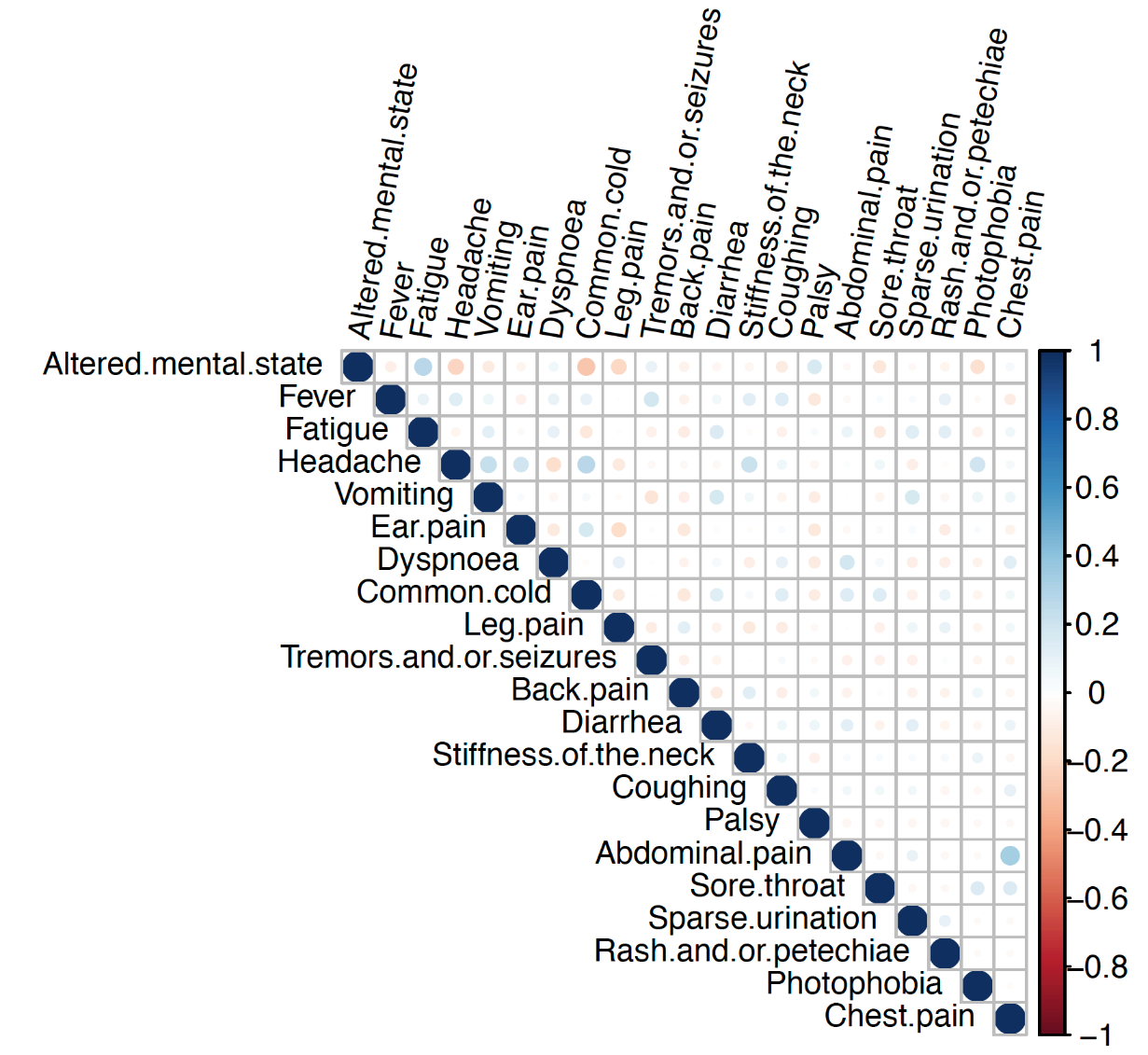


Plot showing paired correlations between symptoms at first contact to emergency medical services for the 209 patients with community acquired bacterial meningitis who had contact to emergency medical services prior to hospitalization between 2016 and 2021 in the Capital Region of Denmark. The highest correlations were seen between abdominal pain and chest pain (r=0.34, CI95%: [0.21;0.45], *P*<0.01), altered mental state and fatigue (r=0.28, CI95%: [0.15;0.40], *P*<0.01), headache and common cold (r=0.27, CI95%: [0.14;0.39], *P*<0.01), headache and vomiting (r=0.24, CI95%: [0.11;0.36], *P*<0.01), headache and stiffness of the neck (r=0.22, CI95%: [0.08;0.34], *P*<0.01) and headache and photophobia (r=0.20, CI95%: [0.06;0.32], *P*<0.01).

**Supplementary Figure 4:** Flow chart of pre-hospital management for the 38 children (A) and 171 adults (B).

A)

B)

Thirty-eight children (A) and 171 adults (B) who had community acquired bacterial meningitis (CABM) called emergency medical services prior to hospitalization. These flow charts illustrate how the patients were handled prior to hospitalization.

**Supplementary Figure 5:** Flow chart of hospital management for the 38 children (A) and 171 adults (B).

A)

B)

Thirty-seven children (A) and 164 adults (B) who had community acquired bacterial meningitis (CABM) arrived at a hospital without being treated prehospitally. These flow charts illustrate how the patients were handled during their hospitalization.
